# Implant-supported mandibular overdentures: a retrospective case series study in a daily dental practice

**DOI:** 10.1186/s40729-021-00345-8

**Published:** 2021-07-08

**Authors:** Shebrina F. Abdoel, Stephanie S. Haagedoorn, Gerry M. Raghoebar, Henny J. A. Meijer

**Affiliations:** 1 General Dental Practice DentalZorg Zaandam, Zaandam, The Netherlands; 2 General Dental Practice Top Dental, Volendam, The Netherlands; 3 Department of Oral and Maxillofacial Surgery, University Medical Center Groningen, University of Groningen, PO Box 30.001, NL-9700 RB Groningen, The Netherlands; 4 Department of Implant Dentistry, University Medical Center Groningen, University of Groningen, Groningen, The Netherlands

**Keywords:** Edentulous mandible, Overdenture, Daily dental practice

## Abstract

**Background:**

Evaluation of dental implant treatment is mostly based on studies with well-controlled study groups treated within a university-based setting. There are no long-term observational practice-based studies known on implant-supported overdentures. The present retrospective study deals with implant survival, peri-implant hard and soft tissue health, surgical and prosthetic aftercare, and satisfaction of patients treated with an implant-supported mandibular overdenture in a daily dental practice.

**Materials and methods:**

Within the years 2006 till 2015, 295 patients were treated with two, three, or four implants for mandibular overdenture treatment in a daily dental practice in Zaandam, The Netherlands. Outcome parameters were scored at a routine yearly inspection including implant loss, plaque index, gingival index, bleeding index, presence of calculus, probing depth, and satisfaction with implant-supported overdenture. Radiographic analysis was performed to assess peri-implant bone changes. Surgical and prosthetic aftercare was obtained from the medical record.

**Results:**

A total of 133 patients were seen for an evaluation visit (mean follow-up 51.2 months). Cumulative implant survival rate in the 2-implant group, 3-implant group, and 4-implant group was 100%, 99.1%, and 97.8% respectively, with a mean peri-implant bone loss of 0.53 mm, 0.61 mm, and 0.40 mm. Patients’ satisfaction was high in all groups.

**Conclusion:**

It was demonstrated, within the limitations of this study, that patients, who were treated with an implant-supported mandibular overdenture in a daily dental practice, experienced a high cumulative implant survival rate and a good peri-implant health, and were very satisfied.

**Trial registration:**

Netherlands Trial Register, NL8867. Registered 15 September 2020—retrospectively registered.

## Background

Edentulous patients often experience problems with their mandibular complete dentures. Lack of stability and retention of their mandibular denture, together with a decreased chewing ability, are the main complaints of these patients [1]. The use of dental implants with a removable overdenture results in an evident increase of patient satisfaction [2, 3]. The survival of 2 or 4 implants in the interforaminal region of an edentulous mandible is over 95% measured over a period of 5–10 years [4–8]. This makes that the recommendation has been proposed that the minimum treatment for complaints concerning a conventional mandibular full denture should be 2 implants with an overdenture [9, 10]. However, also indications for other numbers of implants to support a mandibular overdenture are described. The use of three or four implants is said to be dependent on available bone height, a V-shaped anatomy of the mandible, soreness of the mucosa when loaded, explicit wish for more retention and stability, age of the patient, and certainly also the personal preference of the practitioner. A recent worldwide study revealed that mandibular overdenture treatment is still a frequently executed treatment option, not only in specialized centers, but also in daily dental practices [11].

Reported evaluations of implant treatment are often based on studies with specific, controlled patient groups and mostly carried out within a university setting [12]. Several factors, such as very experienced surgeons, specialized clinics, strict inclusion and exclusion criteria for study participation, and strict attention for hygiene procedures, could influence the outcomes of such studies [13]. Contrary, it appeared that in non-controlled studies in general practices, patients are difficult to motivate to visit aftercare appointments, especially if they have no complaints [14].

For this reason, there is a growing tendency to collect data of implant treatment out of daily dental practices because this possibly better resembles clinical reality [12]. There are no long-term observational studies known out of the daily dental practice with respect to bar-retained mandibular overdenture treatment with two, three, or four implants.

Therefore, the aim of the present retrospective study was to evaluate cumulative implant survival rate, peri-implant health, and patients’ satisfaction with bar-retained mandibular overdenture treatment in a daily dental practice.

## Materials and methods

### Patient enrollment

The study group consisted of fully edentulous patients, formerly complaining about retention and stability of their conventional mandibular denture and treated from 2006 till 2015 in a daily dental practice (DentalZorg Zaandam, Zaandam, The Netherlands) with two, three, or four dental implants, a bar attachment system, and a mandibular overdenture. The design of the study was an observational retrospective evaluation with patients having the overdenture for at least 1 year. There were no inclusion restrictions with respect to general health or smoking habits. At their yearly routine follow-up appointment, all patients were consecutively informed about the study, and they were asked to give verbally informed consent to use their evaluation data. The Medical Ethical Committee of the University Medical Center Groningen provided a waiver as judging the study not subject to the Medical Research Involving Human Subjects Act (Number M21.272914). The study was registered at the Netherlands Trial Register (Number NL8867).

### Surgical and prosthetic procedures

All included patients underwent in the period 2006–2015 in outlines the same surgical and prosthetic procedures. The choice for placing two, three, or four dental implants in the interforaminal region of the mandible was made by a practitioner and patient and based on available bone height, soreness of the mucosa when loaded, age of the patient (in younger patients there was a tendency to place more than two implants because of expected higher functional forces), and explicit wish for good retention and stability of the future overdenture.

The implants were inserted under local anesthesia in the interforaminal region of the mandible. An incision was made on the top of the alveolar process, and a full-thickness flap was raised. In the 2-implant group, the implants were placed in the canine region of the mandible, about 1 cm left and right from the midline. In the 3-implant group and the 4-implant group, there was an equal distance between the implants, and the most lateral implants were placed at least 5 mm medially of the mental foramen. Drilling and insertion of implants (at bone level, two-stage technique) was carried following the standard procedure of the implant brand used (Dyna Helix Implantaatsysteem, Dyna Dental Engineering BV, Halsteren, Nederland). The thickness of the mucosa was adjusted, and the wound was closed with sutures. Patients received antibiotics starting 1 h preoperatively; postoperatively, a 0.2% chlorhexidine mouthwash was used twice daily for 7 days. Patients were advised not to wear the lower conventional denture for 1 week following implant surgery. After that week, sutures were removed, and the denture was adjusted with a soft liner. Eight weeks after implant surgery, second-stage surgery was performed and healing abutments placed. Thereafter, could be started with the prosthetic phase. The prosthetic phase consisted of manufacturing a bar-clip attachment system and a mandibular overdenture. All patients were given a standard oral hygiene instruction for cleaning bar and overdenture. All patients were placed in a yearly routine follow-up program.

### Outcome measures

Outcome measures for the study were collected at the next follow-up appointment. Prior to this visit, patients were informed about the study and asked to participate. Patients who had not been in the dental office for more than a year were asked if they wished an appointment and were thereafter asked to participate.

The following outcome measures were collected:

- Loose and lost implants were scored any time after placement;
- Presence of plaque; the index according to Mombelli et al. [15] was used (score 0: no detection of plaque, score 1: plaque can be detected by running a probe across the smooth marginal surface of the abutment and implant, score 2: plaque can be seen by the naked eye, score 3: abundance amount of plaque);
- Presence of calculus (score 1) or the absence of calculus (score 0);
- To qualify the degree of peri-implant inflammation, the modified Löe and Silness index [16] was used (score 0: normal peri-implant mucosa; score 1: mild inflammation; slight change in color, slight edema; score 2: moderate inflammation; redness, edema, and glazing; score 3: severe inflammation; marked redness and edema, ulceration);
- For bleeding, the bleeding index according to Mombelli et al. [15] was used (score 0: no bleeding when using a periodontal probe, score 1: isolated bleeding spots visible, score 2: a confluent red line of blood along the mucosa margin, score 3: heavy or profuse bleeding);
- Probing depth was measured at 4 sites of each implant (mesially, labially, distally, lingually) by using a periodontal probe (Merit B, Hu Friedy, Chicago, USA); the distance between the marginal border of the mucosa and the tip of the periodontal probe was scored as the probing depth; the highest value per implant was used for the analysis;
- Radiographical mesial and distal bone level change, as measured on the first rotational panoramic radiograph after the osseointegration period and the most recent radiograph of the follow-up appointments; the highest bone loss per implant was used for the analysis;
- For patients’ satisfaction, a questionnaire focused on complaints and consisted of 54 items [17] (Table 5 in Appendix). It was divided into six scales:

A. Nine items concerning functional problems of the lower denture
B. Nine items concerning functional problems of the upper denture
C. Eighteen items concerning functional problems complaints in general
D. Three items concerning facial aesthetics
E. Three items concerning accidental lip, cheek, and tongue biting “neutral space”
F. Twelve items concerning esthetics of the denture

The extent of each specific complaint could be expressed on a four-point rating scale (0, no complaints; 1, little; 2, moderate; 3, severe complaints). Next to this, a general satisfaction score was asked, with a rating from 1 to 10;

- Surgical and prosthetic aftercare was scored any time during the follow-up period.

### Statistical analysis

One observer was responsible for the collection and analysis of all the data. Data were presented as frequencies with, where appropriate, presentation in means or medians.

## Results

In the period 2006–2015, 295 patients received two, three, or four dental implants in the edentulous mandible and were consecutively treated with a bar-clip attachment system an overdenture. The patient characteristics are depicted in Table 1, with a division in patients with two, three, or four implants. The baseline bone atrophy was depicted as the height of the mandible in the canine region and as classification according to Cawood and Howell [18] in the symphysis region.

**Table 1:** Patient characteristics of total group and of patients with two, three, or four implants

	Total group *n*=295	2-implant group *n*=124	3-implant group *n*=86	4-implant group *n*=85
Gender (men/women)	158/137	78/46	41/45	39/46
Mean age in years at implant placement (sd, min–max)	64 (10, 32–88)	62 (11, 32–88)	64 (9, 42–83)	67 (9, 46–86)
Mean edentulous period (years) of mandible before implant placement (sd, min–max)	21 (16, 1–60)	16 (15, 1–60)	21 (16, 1–56)	28 (14, 1–53)
Mean height of mandible in millimeters (sd, min–max)	16 (3.9, 10–25)	17 (3.7, 10–25)	16 (3.7, 10–25)	14 (3.2, 10–25)
Classification according to Cawood and Howell [18] in the symphysis region	Class III, 17% Class IV, 30% Class V, 32% Class VI, 17% Class VII, 4%	Class III, 24% Class IV, 36% Class V, 24% Class VI, 16% Class VII, 0%	Class III, 12% Class IV, 35% Class V, 29% Class VI, 17% Class VII, 7%	Class III, 12% Class IV, 18% Class V, 46% Class VI, 18% Class VII, 6%

Of these 295 patients, 133 patients were actually seen at the follow-up visit. Reasons not to participate were patient died (*n*=5), patient moved to another address and the distance was too far (*n*=14), patient registered at another dental practice (*n*=26), patient indicated that general health is too weak to come to the practice (*n*=54), and patient moved without leaving a new address (*n*=63). No patients had to be excluded because of incomplete data. As part of a strict short-term aftercare protocol, radiographs were taken of each patient at the start of the functional period and served as a reference for peri-implant bone level. The mean follow-up period in the 2-implant group was 49 months (sd 23, min 12, max 104). The mean follow-up period in the 3-implant group was 46 months (sd 20, min 14, max 73). The mean follow-up period in the 4-implant group was 61 months (sd 21, min 12, max 93).

**Fig. 1:**
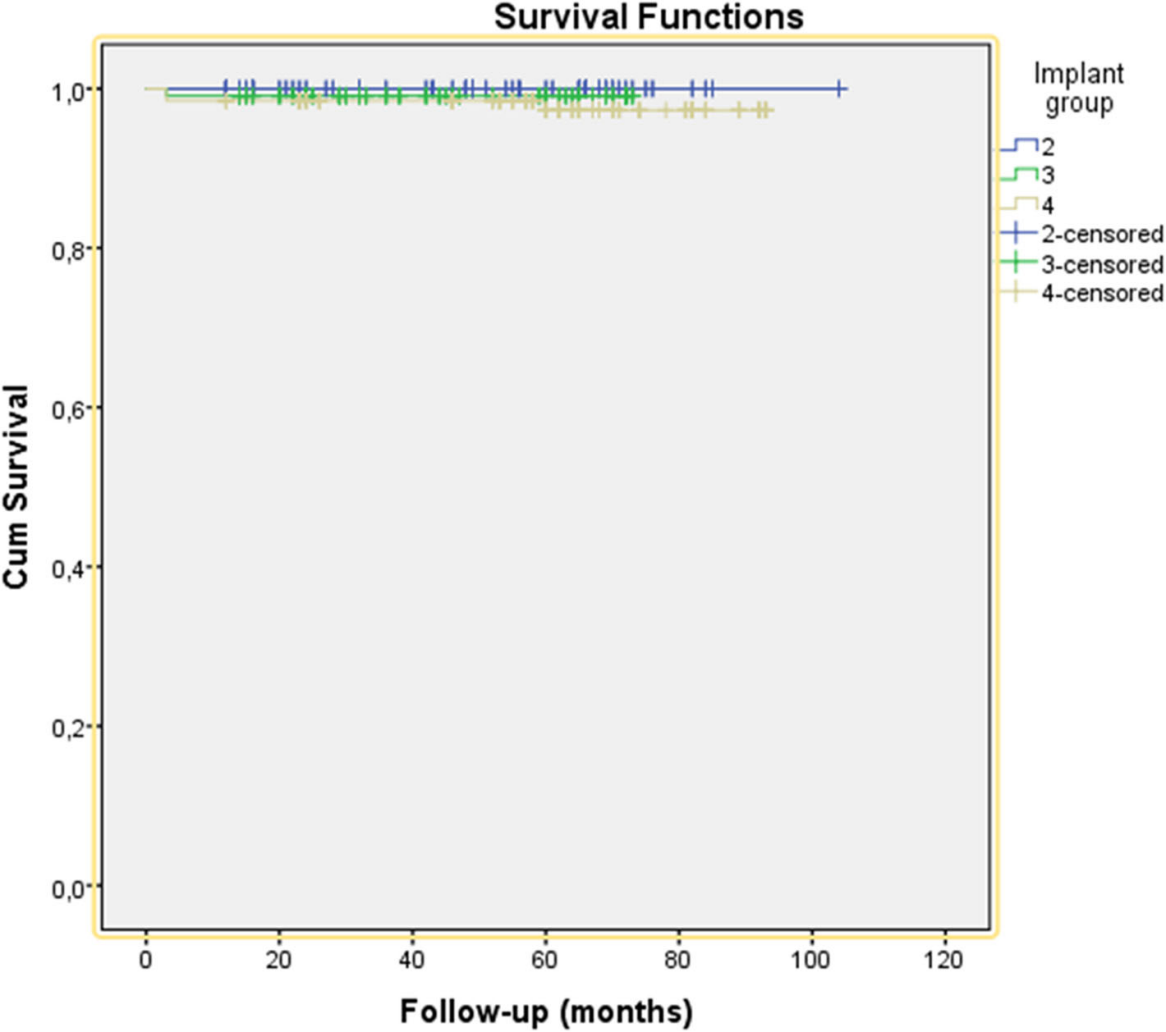
Cumulative survival rate of 2-implant group, 3-implant group, and 4-implant group

In the 2-implant group, no implants were lost: cumulative implant survival 100%. In the 3-implant group, one implant was lost during the osseointegration period: cumulative implant survival 99.1%. In the 4-implant group, three implants were lost (two during the osseointegration period, one after 5 years: cumulative implant survival 97.8%). The cumulative survival rate of the three groups has been depicted in Fig. 1.

**Fig. 2:**
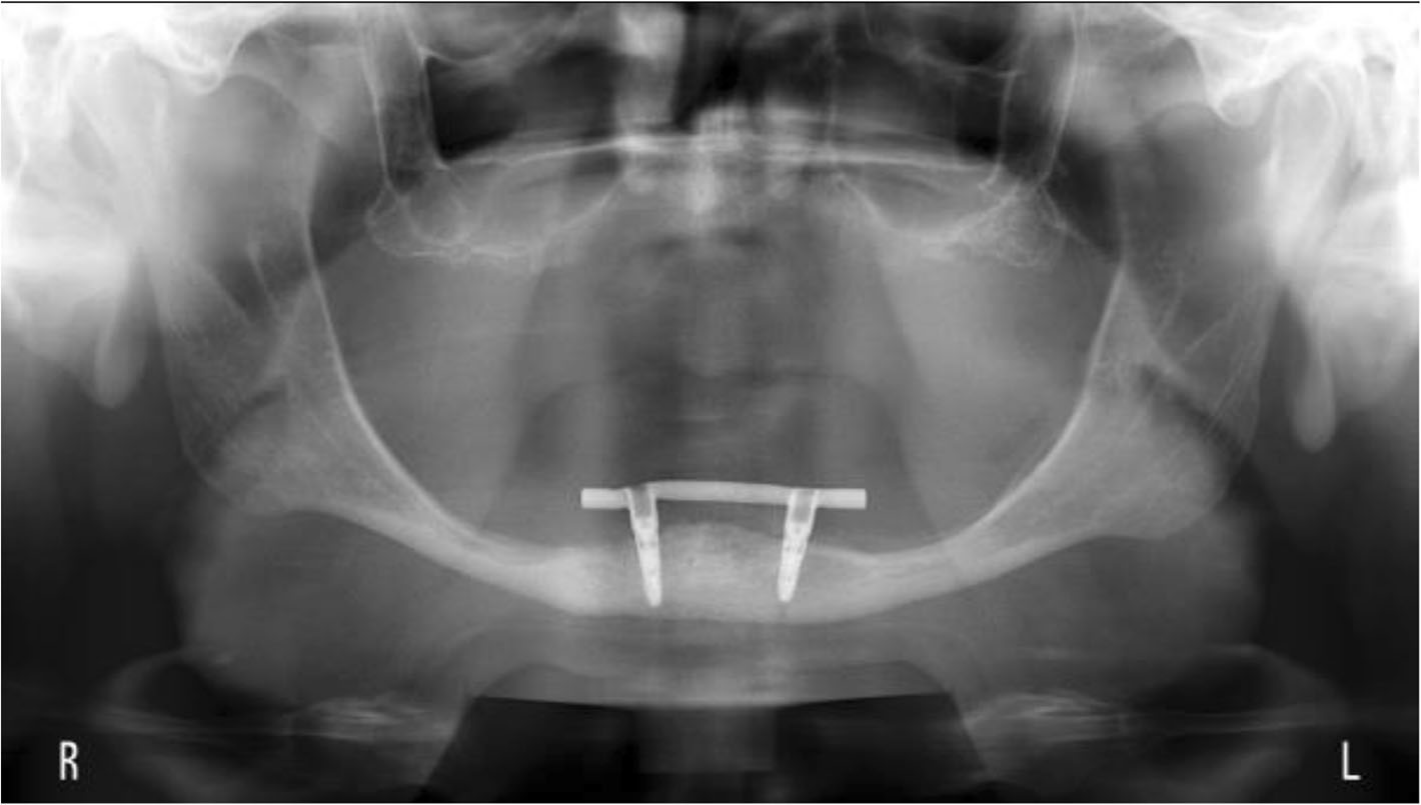
Rotational panoramic radiograph at the time of the evaluation visit of a patient of the 2-implant group

**Fig. 3:**
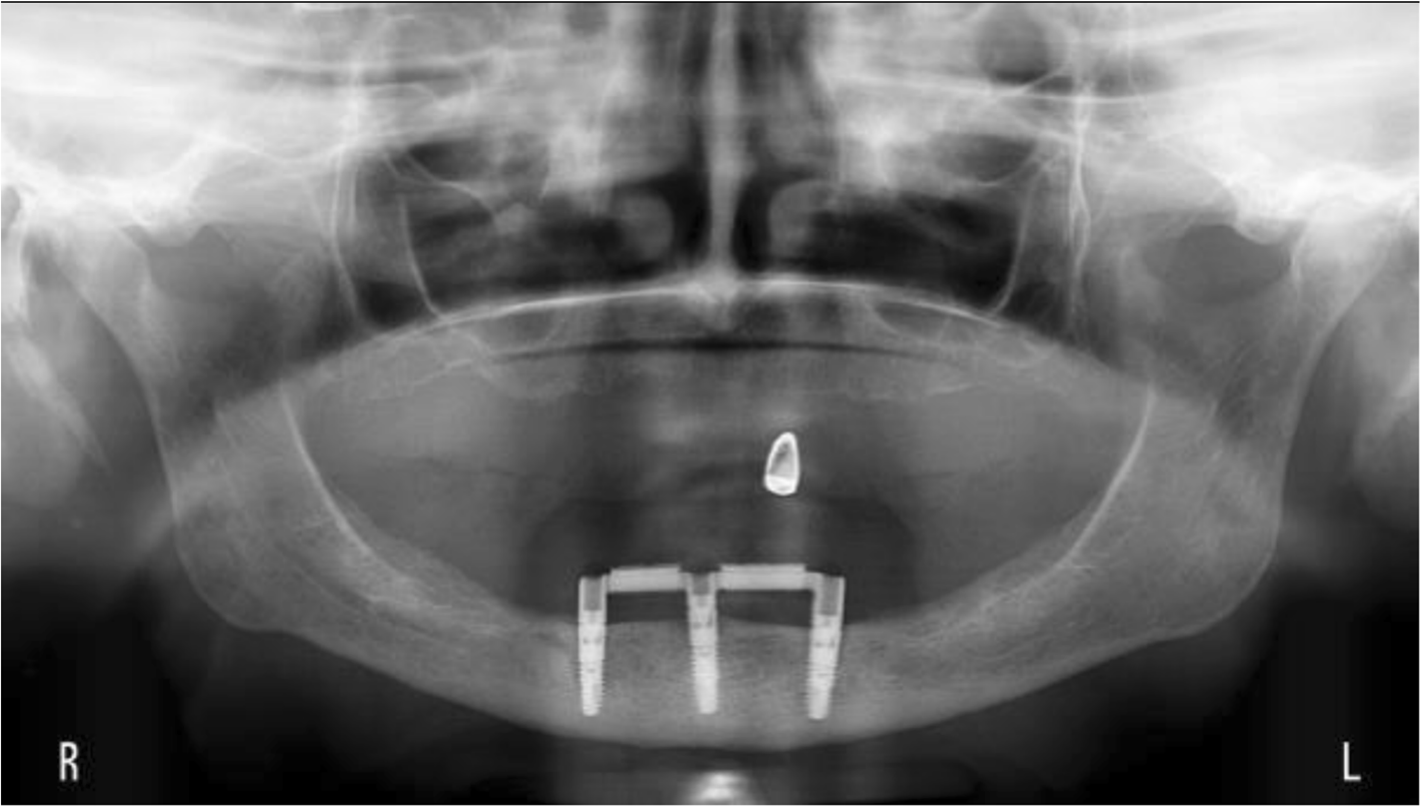
Rotational panoramic radiograph at the time of the evaluation visit of a patient of the 3-implant group

**Fig. 4:**
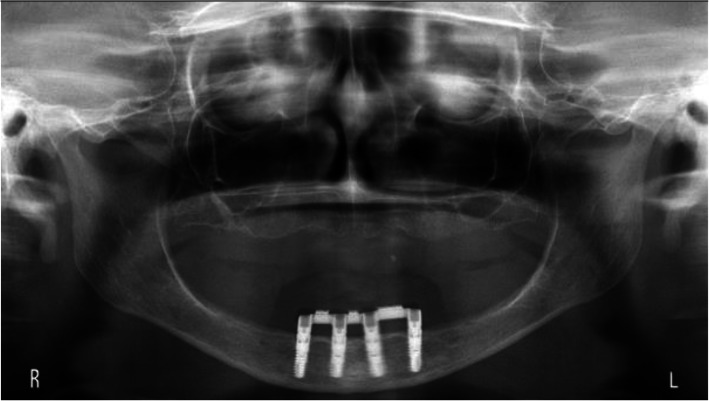
Rotational panoramic radiograph at the time of the evaluation visit of a patient of the 4-implant group

The scores of the indices for plaque, calculus, gingiva, and bleeding were low, hence favorable (Table 2). Mean peri-implant bone level changes were −0.53 mm, −0.61 mm, and −0.40 mm for respectively the 2-implant, the 3-implant, and 4-implant groups (Figs. 2, 3, and 4). The mean scores of the six factors concerning complaints and the general satisfaction score (8.0±1.1, 8.2±0.9, and 8.2±0.8 for respectively the 2-implant, the 3-implant, and 4-implant groups) are listed in Table 3. Surgical and prosthetic aftercare has been listed in Table 4 with number of events as noted in the medical file of the patient.

**Table 2:** Median values with interquartile range of plaque index (score 0–3), calculus index (score 0–1), gingiva index (score 0–3), and bleeding index (score 0–3), and mean values with standard deviation of probing depth (in mm) and peri-implant bone level change (in mm) for each group

	2-implant group *n*=61 patients *n*=122 implants	3-implant group *n*=38 patients *n*=113 implants	4-implant group *n*=34 patients *n*=133 implants
Plaque index [interquartile range]	1 [0;1]	1 [0;1]	1 [0;1]
Calculus index [interquartile range]	1 [0;1]	1 [0;1]	1 [0;1]
Gingiva index [interquartile range]	1 [0;1]	1 [0;1]	1 [0;1]
Bleeding index [interquartile range]	1 [0;1]	1 [0;1]	1 [0;1]
Probing depth (sd)	3.4 (0.9)	3.3 (1.4)	3.9 (1.2)
Peri-implant bone level change (sd)	−0.53 (0.69)	−0.61 (0.60)	−0.40 (0.51)

**Table 3:** Mean score of 6 scales concerning denture complaints (possible range 0–3) and overall satisfaction score (possible range 1–10) at the time of the follow-up appointment

	2-implant group *n*=61 patients	3-implant group *n*=38 patients	4-implant group *n*=34 patients
A. Functional complaints about lower denture (sd)	0.1 (0.2)	0.1 (0.3)	0.1 (0.3)
B. Functional complaints about upper denture (sd)	0.2 (0.5)	0.2 (0.4)	0.2 (0.4)
C. Functional complaints in general (sd)	0.1 (0.3)	0.1 (0.1)	0.1 (0.1)
D. Facial esthetics (sd)	0.1 (0.4)	0.1 (0.3)	0.2 (0.3)
E. “Neutral Space” (sd)	0.2 (0.5)	0.1 (0.3)	0.1 (0.3)
F. Esthetics (sd)	0.0 (0.1)	0.0 (0.1)	0.0 (0.1)
Overall satisfaction score (sd)	8.0 (1.1)	8.2 (0.9)	8.2 (0.8)

**Table 4:** Surgical and prosthetic aftercare (number of events) during follow-up period

	2-implant group *n*=61 patients	3-implant group *n*=38 patients	4-implant group *n*=34 patients
Removal of implant	0	0	1
Placement of new implant	0	0	0
Removal of hyperplasia	1	0	1
Surgical treatment of peri-implantitis	0	0	0
Clip repair	7	0	3
Repair denture base/teeth	14	6	8
Applying temporary soft liner	4	2	1
Relining overdenture	25	14	26
Readjustment occlusion	1	0	1
Bar repair	11	0	1
New bar	1	0	0
New overdenture	3	1	3

## Discussion

Dental implants placed in a daily dental practice to support a mandibular overdenture showed a good survival rate and healthy peri-implant hard and soft tissues, and patients were satisfied after a medium-term follow-up period.

The results from the present study could at best be compared with study groups with a comparable evaluation period, comparable therapy, and treated in a daily dental practice. However, studies with treatment groups out of general practices are lacking, and therefore, it is chosen to compare the present results with study groups treated in a university center or specialist center. Concerning the 2-implant group, clinical scores after a 5-year follow-up period are available of studies of Meijer et al. [19] and Heijdenrijk et al. [6]. These studies reported an implant survival rate of respectively 98.9% and 98.3% and peri-implant bone loss of 1.0 mm and 1.6 mm. The present 2-implant group had 100% cumulative implant survival and a mean peri-implant bone loss of 0.53 mm. Although different parameters are used to express clinical scores, in general, it could be said that comparable healthy peri-implant clinical scores are mentioned (Table 2). Concerning the 3-implant group, there are no comparable studies found in the literature. In the study of Versteegh et al. [20], it was mentioned that the choice for three implants was made if there was no space enough in the interforaminal region to place the initially planned four implants. With respect to stability, a bar-clip attachment system on three implants matches more with a bar-clip attachment system on four implants than on two implants. With two implants, there is a rotation axis, with possible movement of the posterior region of the overdenture around this axis. With three and four implants, there is no rotation axis resulting in a much more stable overdenture. For this reason, results of the 3-implant group and the 4-implant group are compared with the studies of Krennmair et al. [21] and Heschl et al. [22], in which 5-year results are reported of four implants and a bar-clip attachment system. These studies report an implant survival rate of respectively 99% and 98.4% and a peri-implant bone loss of 2.1 mm and 1.0 mm. The present 3-implant group and 4-implant group showed a cumulative implant survival rate of respectively 99.1% and 97.8% and peri-implant bone loss of 0.61 mm and 0.40 mm. Implant survival rates seem to be comparable, but bone loss is less in the present study. An explanation of this could be that the mean edentulous period before implant placement was as well in the 3-implant group as in the 4-implant group more than 20 years (Table 1). In that period, an extensive physiologic resorption of the mandible has taken place. Possibly, part of the peri-implant bone changes in the studies of Krennmair et al. [21] and Heschl et al. [22] could be ascribed to physiologic resorption because implants were inserted in a much more earlier stage. Also, comparison of clinical scores shows a comparable healthy situation (Table 2).

Patient satisfaction and aftercare of as well the 2-implant groups, as the 3-implant group and the 4-implant group, are best to compare with the study of Visser et al. [5]. In this study, a comparison was made between a group with two implants and a mandibular overdenture and a group with four implants and a mandibular overdenture. In both groups a bar-clip attachment system was used. After 5 years of follow-up, the same questionnaires on patient satisfaction were used and also the same method to analyze aftercare. All six satisfactions in as well the study of Visser et al. [5] as in the present study in all three groups show a mean score less than 0.5 (on a scale of 0–3), meaning a high patient satisfaction (Table 3). With respect to aftercare during the 5-year follow-up, it is remarkable that “Repair denture base/teeth” and “Relining overdenture” are the most present in all three groups. An explanation for fracture of base and teeth could be a thin denture base in the anterior region and limited space to attach teeth in the acrylic resin. A bar is housing within the acrylic resin of the overdenture and at cost of a substantial amount of acrylic resin. This could result in less strength. “Repair denture base/teeth” has also been mentioned in the study of Visser et al. [5]. Apparently, this is an inherent complication in overdenture therapy. In the study of Visser et al. [5], only limited “Relining overdenture” was scored. An explanation for this is not obvious. In both studies, the included patients were edentulous for a long period, so ongoing physiologic resorption could not be the reason. In the present study, also the item “Bar repair” was scored a number of times in the 2-implant group, whereas this did not happen in the study of Visser et al. [5]. Further analysis learned that in the present study sometimes distal bar extensions were used to limit rotation of the overdenture. These extensions appeared to be prone to fracture.

Overall comparison of outcomes of the present study with outcomes in the literature demonstrates that results of mandibular overdenture therapy in the daily dental practice are as good as in a university/specialist center.

A limitation of the present study is that no reasonable statistical comparison can be made between the 2-implant group, the 3-implant group, and the 4-implant group. The initial situation was not the same for each patient. The choice for the number of implants was in the period 2006–2015 not only based on the height of the mandible, but also on the complaints the patient had and possibly the explicit wish of the patient. Particularly, the explicit wish of the patient for a specific number of implants could have influenced the treatment plan and therefore the patients’ satisfaction in the end. Another limitation of the study is the fact that a large number of patients did not participate in this retrospective evaluation. Of the initial 295 included patients, 133 could be evaluated. This is only 45%, whereas in university-based studies often above 90% is achieved. In prospective controlled studies, patients are at the beginning explicitly asked to participate and sign an informed consent. The retrospective design of the study could be a reason not to participate. Another reason not to participate is possibly a lesser bond after therapy between patients and practitioners in a general dental practice; people do not come back for control visits or choose to attend another practice. The assumption that not participating is independent from the state of health of the implants and the satisfaction of patients can be made, but may also be questioned.

## Conclusion

Within the limitations of this study, it has been demonstrated that patients treated with an implant-supported mandibular overdenture in a daily dental practice experienced a high cumulative implant survival rate and a good peri-implant health, and were very satisfied.

## Data Availability

The datasets used and/or analyzed during the current study are available from the corresponding author on reasonable request.

## Appendix

**Table 5:** Questions of patient satisfaction questionnaire divided into six scales (A, functional problems of the lower denture; B, functional problems of the upper denture; C, functional problems complaints in general; D, facial esthetics; E, accidental lip, cheek, and tongue biting “neutral space”; F, esthetics of the denture)

	Question	Scale
1	Upper denture gets loose during eating	B
2	Upper denture gets loose during speaking	B
3	Upper denture gets loose during yawning	B
4	Upper denture hurts when eating hard food	B
5	Upper denture hurts when eating soft food	B
6	Upper denture hurts when eating granular food	B
7	Upper denture fits badly	B
8	Lower denture fits badly	A
9	Lower denture gets loose during eating	A
10	Lower denture gets loose during speaking	A
11	Lower denture gets loose during yawning	A
12	Lower denture hurts when eating hard food	A
13	Lower denture hurts when eating soft food	A
14	Lower denture hurts when eating granular food	A
15	Lips have fallen in	D
16	Cheeks have fallen in	D
17	Mouth has fallen in	D
18	Burning sensation under the upper denture	B
19	Burning sensation under the lower denture	A
20	Teeth are too big	F
21	Teeth are too small	F
22	Teeth are too white	F
23	Teeth are too dark	F
24	Teeth are too far forward	F
25	Teeth cannot be seen enough	F
26	Teeth are too obvious	F
27	Teeth click while eating	C
28	Teeth click while speaking	C
29	Tongue biting	E
30	Cheek biting	E
31	Lip biting	E
32	Food gets under the lower denture	A
33	Food gets under the upper denture	B
34	Chewing takes too much time	C
35	Food is difficult to chew	C
36	Teeth are not in the position I would like	F
37	Teeth are not straight enough	F
38	Dentures are not alike my natural teeth	F
39	Other people see I have dentures	F
40	An agglutinant is needed for retention	C
41	The denture does not look good on me	F
42	Taste is not sufficient	C
43	Speaking is not clear	C
44	Not enough room for the tongue	C
45	Gagging reflex because of the denture	C
46	Swallowing problems	C
47	Denture rattles	C
48	Nervousness because of the denture	C
49	Denture becomes dislodged during laughing	C
50	Full sensation due to the denture	C
51	Dry mouth	C
52	Too much saliva	C
53	Denture-sucking habit	C
54	Denture tightens	C

## Abbreviations

n: Number
sd: Standard deviation
min: Minimum
max: Maximum
